# A Novel Multidimensional Tensile, Shear, and Buckling Sensor for the Measurement of Flexible Fibrous Materials

**DOI:** 10.3390/s24020406

**Published:** 2024-01-09

**Authors:** Liang Luo, George Stylios

**Affiliations:** Research Institute for Flexible Materials, Heriot Watt University, Edinburgh EH14 4AS, UK

**Keywords:** low-stress measurement, tensile, shear, buckling, fabric, FE, simulation, beam theory, multi-sensor

## Abstract

To meet the complex and diverse demands for low-stress mechanical measurements of fabrics and other flexible materials, two integrated multidimensional force sensors with the same structure but different ranges were explored. They can support both rapid and precise low-noise, high-precision, low-cost, easy-to-use, reliable, and intelligent solutions for the complex measurement of fabric mechanics. Having analysed the mechanical relationship of the parallel beam theory, and considering the specific requirements of fabric measurement, a novel multi-dimensional force sensor is designed, capable of measuring tensile, shear, and buckling properties. Finite element analysis is used to simulate the mechanical performance of this sensor for fabric-loading/unloading measurement, and the sensitivity of the mechanical quantity transfer, the amount of sensor deformation, the stress distribution, and the degree of inter-dimensional coupling have been investigated and verified. The basis for subsequent digital processing is achieved by a low-offset, low-temperature-drift, low-power-consumption analogue front end, 24-bit ADC circuit, and signal conditioning electronics, suitable for the measurement of fabric mechanics under low stress, which is like the end-user requirements. The sensor information channel is supported by a host microcontroller with a DSP and a floating-point processing instruction set. Information processing is performed in time-sharing with the support of a multitasking real-time operating system. The purpose of designing this sensor is to facilitate the development of a new testing instrument, which will adopt the advances of current instruments whilst eliminating their shortcomings.

## 1. Introduction

### 1.1. Historical Background of Fabric Instrumentation

Yousef and Stylios reported that Haas in the 1900s was the first to define fabrics as engineering materials and used models to determine the behaviour of the outer shell structure of spaceships during the Zeppelin era [1]. In the 1930s, Peirce realised the desirability of designing physical tests to analyse and reflect the sensation of cloth touching [2,3,4]. They developed physical measurements for calculating fabric bending, compression, and frictional properties, and used quantitative criteria to assess fabric quality. 

Continued research on low-stress fabric mechanical properties became important in the 1960s and ways to measure bending, shear, and tensile properties were introduced by Lincoln, Behre, and Dahlberg [5], and by Cusick [6]. Buckling measurement was first applied to clothing fabrics by Grosberg and Swani [7]. Bending, shearing, and stretching were further explored for the sewability of fabrics by Stylios et al. [8,9,10], and the shape, fit, and drape ability of garments by Postle [11].

Around 1969, as part of a fundamental study of fabric mechanics, Kawabata and his team began preliminary investigations on fabric handle. The Hand Evaluation and Standardization Committee (HESC) was established in 1972 [12]. Kawabata and Niwa started to clarify and understand and quantify fabric hand evaluation [13]. On this basis, the Kawabata team in Japan and their counterparts in CSIRO, Australia commercialised some cloth instrument measurement devices, such as the KES-F test system [14] and the FAST test system [15], respectively. Among them, the KES-F test system provides complete loading and unloading fabric measurement curves, whilst the FAST data provide points from which properties can be estimated. These instruments are used by many universities and scientific research associations, as well as by companies, such as Marks and Spencer Plc, Unilever Plc, Toray, Toyobo, and Coats Viyella.

After the commercialisation of the KES-F and FAST test systems, Stylios developed a combined automated testing system FAMOUS whilst at Bradford University (1995–2001) [16], and SDL Atlas Ltd. has commercialised the FTT fabric tactile tester [17]. Software developers of 3D CAD, such as Browzwear (https://browzwear.com/blog/see-fabrics-true-life-3d-browzwears-new-fabric-analyzer, accessed on 7 September 2018), have also commercialised, in combination with their CAD, a fabric-testing device (FAB) for their own garment simulation software, but it is a very simplified version of FAST without the capability to measure recovery and shear [18], as is also the case for Optitex [19] and Clo3D [20]. The commercialisation of these fabric-testing devices is a testament of the market demand for fabric measurement. Despite these efforts, however, none of these instruments can provide a complete loading/unloading curve of low-stress mechanical fabric properties, except for the KES-F and FAMOUS. For fabric mechanics modelling and simulation, it is very important to obtain complete loading and unloading curve information of fabric mechanical properties. The key to effective fabric measurement is the precise sensor design and use, and this is the focus of this paper.

### 1.2. Evaluation of the KES-F System

There have been many literature reviews, evaluations, and uses of the KES-F measurement system [21,22,23].

The main instrumentation shortfalls are as follows:

1. The fabric clamps for tensile and shear measurement are connected to the instrument support by friction. The advantage of this construction is that the sensor is not subjected to the forces involved in operating the fabric clamps and is not affected by the gravity of the fabric clamps themselves. The disadvantage is that frictional forces are introduced during measurement and lead to measurement errors, especially when it is at low force.
2. During shear measurement, the shear directional force component formed by the pre-stretch force applied to the fabric is mistakenly measured as shear when the fabric shear angle is not equal to zero. This mechanical component was not removed in the measurement processing circuit (the KES-F used an analogue circuit to process the signal and, based on the technology available at the time, this mechanical component could have been removed by the operational amplifier circuit).
3. Dynamic pre-tension for tensile and shear measurement is not supported.
4. There is a need for the instrument to provide digital, intelligent, and networking abilities for end-user requirements.

In recent years the KES-F has become the automated KES-Auto and the operator error may have been eradicated, but the system is very expensive and is not supported by customer service in most countries outside Japan, leaving most small- to medium-size textile companies without a reliable measurement of fabric mechanics.

FAMOUS is designed and built to overcome all the above; however, it is not being commercialised despite being used in research and new product development by companies such as Jhonson Controls, W L Gore, Camira Fabrics, etc. [24].

## 2. Machine Instrumentation for Testing

### 2.1. The Measurement of Fabric, and Its New Requirements

The requirements for measuring the low-stress mechanical properties of fabrics are diverse and complex. These needs have different expectations for the accuracy of measurement results, the operability and convenience of the measurement process, low costs, and the reliability of instrument use. 

**Figure 1:**
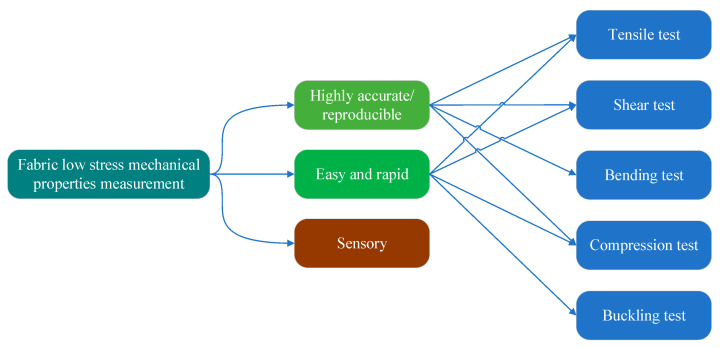
Fabric low-stress mechanical property measurement classification diagram. The KES performs tensile, shear, bending, and compression; the FAMOUS performs tensile, shear, buckling, and compression; our proposed design performs in rapid mode—tensile, shear, buckling, and compression, and in precise mode—tensile, shear, bending, and compression.

As shown in Figure 1, a sensor is used for fabric mechanical property measurement using standard samples and performing quick testing, at low cost, or precise testing, and conveniently obtaining measurement results through a data comparison between the fabric under testing and a standard sample. One way to provide digital support for the sensory measurement is to develop mobile applications for it and connect applications with a fabric cloud database [25]. 

**Figure 2:**
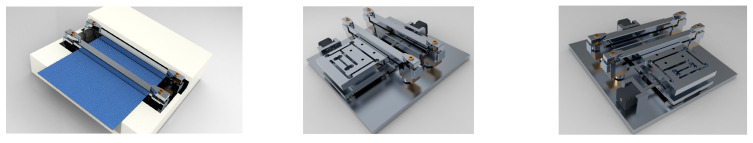
Multifunctional fabric mechanical properties rapid measurement solution—instrument main diagram, showing the embodiment of the clamps and the sensor head (see also the relationship between sensor and clamps in Figure 10).

With the advent of the digital age, the industry is facing the challenge of digitalisation. Stylios [25] and Hearle [26] argued that the textile and apparel industry faced challenges from empirical craftsmanship to engineering design. Fabric and apparel designers who use the software need to always have instruments on their desks for the rapid measurement and evaluation of fabrics. Such an instrument to them is like a multi-meter to an electrical engineer. But the instrument must also provide adequate measurement accuracy and data integrity (with complete load and unload curve measurement capabilities) and be able to support network capabilities. This is the application scenario of our proposed fabric measurement solution, for rapid, easy, and precise fabric measurements (see Figure 1). To meet the needs of rapid measurement application scenarios, the instrument uses buckling measurement instead of pure bending measurement. The complete (nonlinear elasticity and hysteresis) information of pure bending measurements is extracted from the data of buckling measurements by machine-learning software. The measurement range of tensile is also reduced compared to KES-F. On the one hand, it balances the different demands on the sensor measuring range for tension measurement and buckling measurement. On the other hand, it reduces the driving power requirement for the measurement process. At the same time, the instrument structural design requirements are simplified. This solution supports tension, shear, and buckling measurements using an integrated multi-dimensional sensor especially developed for it. A schematic of the embodied testing instrument is shown in Figure 2. The compression measuring unit and the surface properties measuring units are not shown in the figure. 

The third-level category of fabric measurement is the requirement of high-precision measurement fully compatible with the KES-F system. The high-precision measurement solution has the capability of pure bending measurement and a larger range of tensile measurement. The instrument is digitalised and has a virtual measurement and control interface. The management of measurement data is performed by a front-end database, and the software provides component decomposition and data compression functions for mechanical properties. A database also supports data-sharing functions, which can easily convert between different mechanical units.

The above two fabric instrument measurement solutions require two integrated multi-dimensional sensors with the same structure, dimensions, installation method, and measurement principle, but different measuring ranges. They are called rapid measurement multi-dimensional sensors and precise measurement multi-dimensional sensors, respectively. This paper focuses on the exploration of their design and their finite element simulation.

The need for two textile instrumentation measurement solutions dictates the multidimensional nature of the sensor. Measurements of tension and buckling belong to the same dimension. The direction of the shear measurement is perpendicular to the direction of the tensile measurement and belongs to another dimension. Therefore, measurements of tensile and buckling require sensors in both dimensions. When the fabric undergoes shear deformation, the orthogonal relationship between the warp and weft yarns of the woven fabric will be disturbed. The shear force and the tensile force along the yarn direction are no longer perpendicular. However, their forces can still be obtained by transforming the measurements of two mutually perpendicular forces. In the KES-F system, two independent sensors are used to measure this force in two dimensions. Its sensors are laid out in such a way that measurements are affected by friction. At the same time, the measurement method of KES-F also makes the structure of the instrument very complex, an important reason why these instruments are expensive. In addition, this structure also makes it difficult to integrate other measurement functions into a unified platform. Moreover, this also makes the instrument operation complex and time-consuming. This specially designed multi-dimensional sensor completely avoids these problems, and the instrument structure becomes simple, and other measuring provisions can be easily integrated into the unified platform, and instrument costs can be significantly reduced. All measurements can be completed in one operation, and the entire measurement system can be reduced in size. 

The modularity, integration, and intelligence of sensor units enable them to better adapt to the needs of the solution. Solutions require both high-precision measurement results and low-cost manufacturing. Traditional sensor design concepts often find it difficult to consider these two requirements. The cost of traditional high-precision sensors and their analogue front ends is mainly consumed in the analogue calibration and adjustment of the measurement output. Advances in semiconductor technology have provided applications with low cost and powerful embedded microcontrollers and high-precision integrated analogue front ends and make it possible to apply digital calibration. Multidimensional sensors usually suffer from coupling interference problems between dimensions, and these interferences need to be effectively eliminated. Machine-learning algorithms such as support vector machines are effective ways to eliminate interference between dimensions. Fabric measurement also involves some dynamic transformation and measurement correction calculations that need to be processed. Unified processing of these computing needs by analogue front end and microcontroller integration in the sensor module can minimise the transmission distance of the analogue signal, thereby reducing noise interference. The entire sensor becomes an integrated intelligent module, which implements control signal input and information, and status output through standard interfaces. 

The sensor is also interfaced with motor control, via a virtual instrument measurement and control interface, eliminating the need to use a control box like the KES-F system. In addition, we have designed each module with low energy so that the instrument can be powered by a USB interface or battery. The application of these technologies not only reduces the manufacturing cost of the instrument, but also promotes the miniaturisation of the fabric-measuring instrument so that it can be conveniently placed on the user’s desk.

The design and working principle of the measurement instrument which has, as its core, this sensor will be reported in another paper, but the design, construction, and validation of the sensor will be carried out by theoretical physics (parallel beam) and by simulation (FE), which are reported next. 

### 2.2. Sensor Measurement Range

To be compatible with the KES-F instruments, the fabric sample size was chosen as 200 mm × 200 mm.

The measurement range of integrated multi-dimensional force sensor for the rapid measurement solution is:

- Fabric tensile force 0 to 10 N
- Fabric shear force −10 N to 10 N
- Fabric buckling force 0 to 10 N.

Compatible with the KES-F system measuring range, the measurement range of the integrated multi-dimensional force sensor for the precise measurement solution is:

- Fabric tensile force 0 to 100 N
- Fabric shear force −10 N to 10 N

The above-mentioned shear and buckling force measurement ranges are larger than the shear and buckling forces that the sensor may be subjected to during fabric tests. The reason for taking the large measurement range value is the need to make a compromise between measurement accuracy and the effect of the dead weight of the fabric clamp on the sensor; in addition, with a reasonable analogue front-end design, the strain sensor can obtain a force resolution of 1 mN in the above measurement range. The selection of the tensile force measurement range of the sensor considers the buckling measurement sensitivity needed in order to simplify the structure of the sensor and processing circuit. This measurement range already meets the needs of garment and fabric simulation, but, most importantly, it is compatible with real garment-processing and weaving conditions.

In the next section of this paper, we show how, using the analysis of the mechanical relationship of the parallel beam sensor, we constructed the design of two elastic elements fully integrated that can achieve multi-dimensional measurement, along with the strain gauge bridging schemes of both elements. The theory of elasticity [27] is employed to calculate the dimensions of the sensitive locations of the two elastic elements, and then we performed finite element simulation. The feasibility and application of the elastic elements of this sensor are verified, and the main sources of inter-dimensional coupling are analysed. The strain utilisation factor and the sensitivity of the sensor at the critical locations of the elastic elements are estimated. 

## 3. Sensor Design

Here, we will investigate and discuss a novel multi-dimensional sensor.

### 3.1. Sensor Transformation Chain

**Figure 3:**
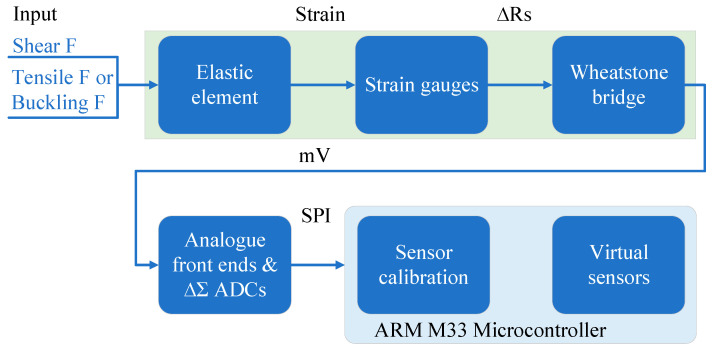
Structure and information flow diagram of intelligent integrated sensing module.

There are many ways to convert a measured mechanical quantity into an electrical signal. Because the strain-sensing method has the characteristics of high conversion accuracy, low price, ease of use, and being multi-dimensional, it is selected as the measurement method of fabric mechanical properties in this paper. The structure and information flow relationship of the sensor module is shown in Figure 3. Strain sensors are parametric. The sensor senses the force through its elastic element and transforms the force into the corresponding strain. The strain at the sensitive position of the elastic element is converted by the strain gauge attached to its surface into the corresponding change in the resistance value of the strain gauge. Then, a Wheatstone bridge composed of strain gauges converts the change in the resistance value of the strain gauges into a corresponding analogue voltage output.

To simplify the structure of the analogue signal conditioning circuit and maximise device integration and digital intelligence, a high-speed, high-precision multi-channel synchronous-sampling 24-bit ADC is chosen as the analogue front end of the sensor. The analogue front end interfaces with the microcontroller host through the SPI serial communication interface.

The host is an ARM Cortex-M33 microcontroller with an extended instruction set for floating-point operations and an extended instruction set for DSP. ARM Cortex-M33 is a 32-bit RISC microcontroller core licensed by ARM. The above-mentioned extended instruction set of the microcontroller is very important for the real-time data processing of the sensor module and virtual sensor calculation process. In the microcontroller, sensor calibration and virtual sensor functions are handled in real time as tasks through scheduling and management by its real-time operating system.

### 3.2. Elastic Elements Base Structure Analysis

One of the challenges of sensor design comes from the need for the sensor to have high noise immunity, so it is only sensitive to the force to be measured and shows the greatest possible suppression ability to the interference of forces in other directions and other physical quantities. In our case, integrated sensing modules need to measure forces from two orthogonal directions simultaneously. The two forces to be measured interfere with each other. For fabric precise measurement, the force range in the tensile direction is ten times that of the force in the shear direction. In addition, since the fabric clamp is completely supported by the sensor, the weight of the clamp also affects the sensor. These challenges need to be properly addressed through design.

The design of the elastic element is the most important part of the sensor. Due to its superior resistance to lateral force interference, the parallel beam elastic element was chosen [28] as the basic configuration for the integrated fabric force sensor. Duralumin-copper alloy with a low elastic modulus is chosen for making the elastic elements.

**Figure 4:**
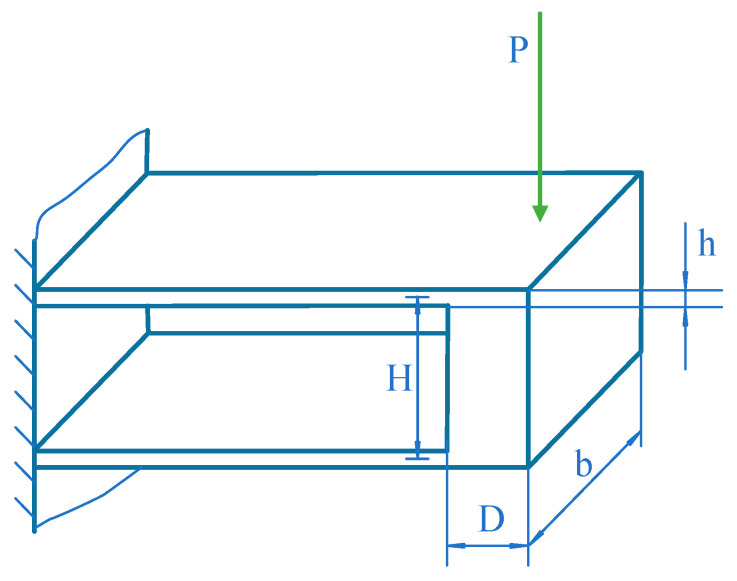
Parallel beam structure diagram.

As shown in Figure 4, the parallel beam elastic element belongs to a three-order statically indeterminate structure. Let the thickness of the parallel beam be h and the width be b. The distance between the parallel beams is H. The thickness of the connecting members between the parallel beams is D. The dimension of D is chosen to be much larger than h, and the connection between parallel beams can be approximated as a rigid connection.

**Figure 5:**
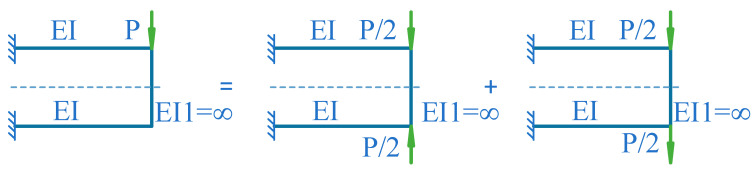
Decomposition diagram of parallel beam elastic body.

To simplify the calculation process, the perceived force *P* of the elastic element can be decomposed into a pair of co-directional forces composed of *P/2* and a pair of opposite forces composed of *P/2* according to Figure 5. The pair of opposite forces are cancelled due to the connecting members between parallel beams treated as rigid bodies.

**Figure 6:**
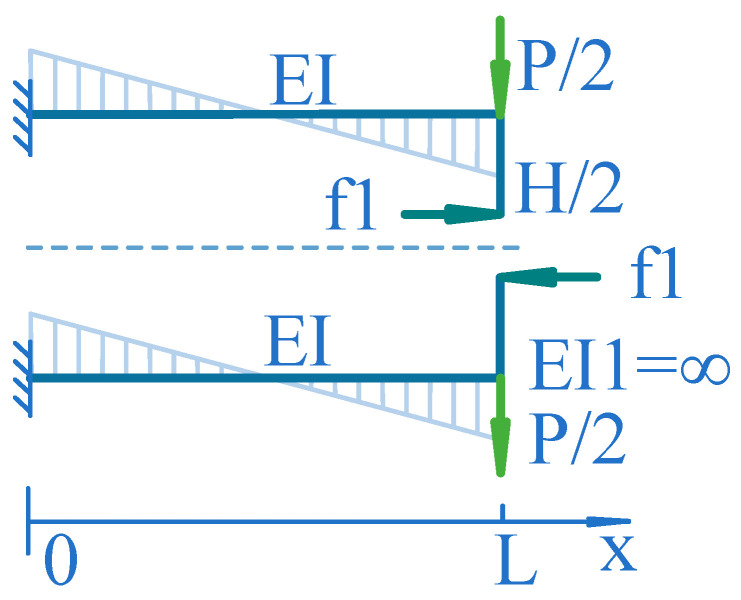
Primary structure force analysis diagram of a parallel beam elastic element.

As shown in Figure 6, if the external torque on the connecting rigid body between parallel beams is zero, assuming that the connection between the two parallel beams is cut along the symmetry axis of the parallel beam elastic element, the primary structure of the elastic element is obtained. The corner θ formed by *P/2* on the upper cut plane is:

(1) $$\begin{array}{c} \theta =\frac{P}{2EI}\int_{0}^{L}\left(L-x\right)dx=\frac{PL^{2}}{4EI} \end{array}$$

where $I=\frac{bh^{3}}{12}$ is the moment of inertia of the beam section, *E* is the material elastic modulus of the beam, and *L* is the length of the upper parallel beam.

The corner $\theta_{1}$ formed by $f_{1}$ on the upper cut plane is:

(2) $$\begin{array}{c} \theta_{1}=\frac{M_{1}}{EI}\int_{0}^{L}dx=\frac{M_{1}}{EI}L \end{array}$$

Let $\theta -\theta_{1}=0$, i.e., we can obtain:

$M_{1}=\frac{P}{4}L$, which is:

(3) $$\begin{array}{c} f_{1}=\frac{P}{2H}L \end{array}$$

**Figure 7:**
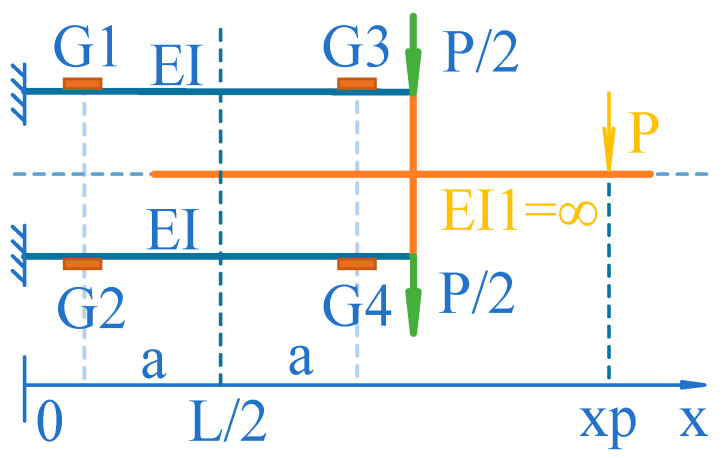
Schematic diagram of the relationship between the strain of the parallel beam and the point of application of the sensing force.

As shown in Figure 7, it can be proven that, when the action point of the *P* force is translated to $x=\frac{L}{2}$, $f_{1}=0$, the parallel beam elastic element transforms into an S-type elastic element. Compared with *P* acting on *x = L*, the *P* force exerts a negative torque on the rigid connection between parallel beams, and its value is $\frac{PL}{2}$. The radius of curvature *r* of a parallel beam along the x-direction can be expressed as:

(4) $$\begin{array}{c} \frac{1}{r}=\frac{d\theta }{dx}=\frac{M\left(x\right)}{EI} \end{array}$$

where *M(x)* represents the force couple of the parallel beam at the x position. When the force couple causes bending, the intermediate layer length of the beam, dx=rdθ, does not change. The length of the outer layer of the beam is:

(5) $$\begin{array}{c} dl=\left(r+\frac{h}{2}\right)d\theta \end{array}$$

According to the definition of strain, the surface strain ε of a parallel beam is:

(6) $$\begin{array}{c} \varepsilon =\frac{dl-dx}{dx}=\frac{h}{2r}=\frac{M\left(x\right)}{EI}\;*\;\frac{h}{2}=\frac{M\left(x\right)}{EW} \end{array}$$

Among them, $W=\frac{I}{\frac{h}{2}}=\frac{bh^{2}}{6}$ is the section modulus of the beam. Since $M\left(x\right)=\frac{P}{4}\left(L-2x\right)$, so the strain $\varepsilon_{m}$ at the G1 position is:

(7) $$\begin{array}{c} \varepsilon_{m}=\frac{Pa}{2EW} \end{array}$$

When the action point of the *P* force is at $x=\frac{L}{2}$, the magnitudes of the strains at positions G1, G2, G3, and G4 are equal; G1 and G4 are positive (tensile) strains, G2 and G3 are negative (compressive) strains, and vice versa. The strain induced by the elastic element at the strain gauge leads to a change in the resistance of the strain gauge. 

Let the sensitivity coefficient of the strain gauge be *k*, and the corresponding strain gauge resistance change is:

(8) $$\begin{array}{c} \frac{∆R}{R}=k\varepsilon_{m} \end{array}$$

**Figure 8:**
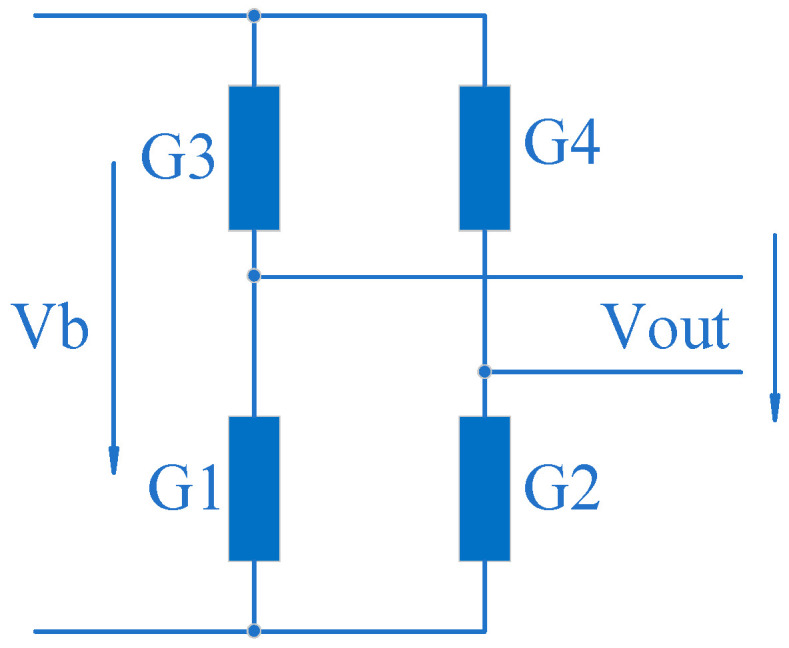
Measurement bridge consisting of strain gauges.

A Wheatstone bridge consisting of strain gauges is shown in Figure 8. The sensitivity coefficient *K* of the sensor is:

(9) $$\begin{array}{c} K=\frac{\left(1+k\varepsilon_{m}\right)R}{2R}-\frac{\left(1-k\varepsilon_{m}\right)R}{2R}=k\varepsilon_{m} \end{array}$$

When the stress point of the elastic element is offset by the distance d of the *L/2* point along the x-direction, it is equivalent to exerting a torque Pd on the rigid connection of the parallel beam. This torque needs to be balanced by another torque. They have a balanced relationship:

$Pd=f_{1}H$. That is, $f_{1}=\frac{Pd}{H}$. $f_{1}$ produces a corresponding strain in the elastic element. According to Hooke’s law, its value is $\varepsilon_{d}=\frac{f_{1}}{Ebh}$. That is:

(10) $$\begin{array}{c} \varepsilon_{d}=\frac{Pd}{EbhH} \end{array}$$

At G1 and G3, this strain is superimposed on the original strain in a positive direction. In G2 and G4, the strain is superimposed on the original strain in the negative direction. Although the distribution of strain on the elastic element changes due to the offset of the force point, it, ideally, has no effect on the output due to the suppression of the Wheatstone bridge. That is, the sensor is not sensitive to the point of application of the force. 

When the parallel beam sensor is subjected to tension or pressure in the x-direction, corresponding strains are generated on the upper and lower half arms of the parallel beam, which, ideally, has no effect on the output, meaning that the sensor is not sensitive to lateral forces.

Although, ideally, parallel beam sensors are not sensitive to disturbing forces, disturbances may still appear in the sensor’s output signal due to manufacturing errors. Therefore, it is still necessary to carefully optimize the anti-interference ability of the sensor in the structural design and during the sensor manufacturing process. For high-precision measurement, it is even more important to perform real-time online correction of the sensor output through virtual sensor technology.

To maximise the stiffness of the sensor, the strain on the sensor elastic element should be concentrated as much as possible in the area where the strain gauges are attached. The rest of the elastic element should be as structurally stiff as possible. The parallel beam elastic element naturally evolves into a double-hole parallel beam elastic element.

### 3.3. Sensor Structural Design

**Figure 9:**
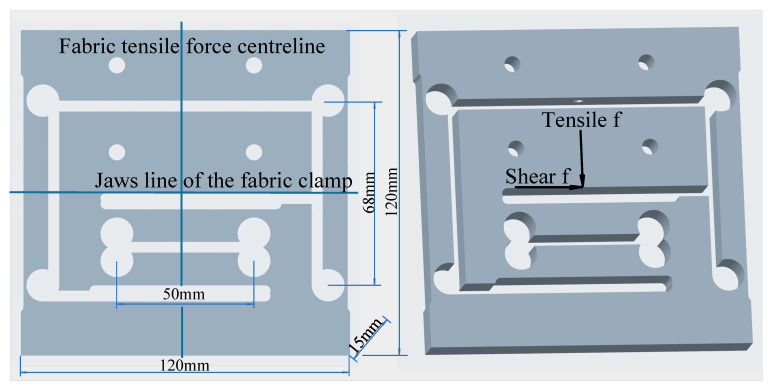
Structure of the elastic elements of the multi-sensor.

**Figure 10:**
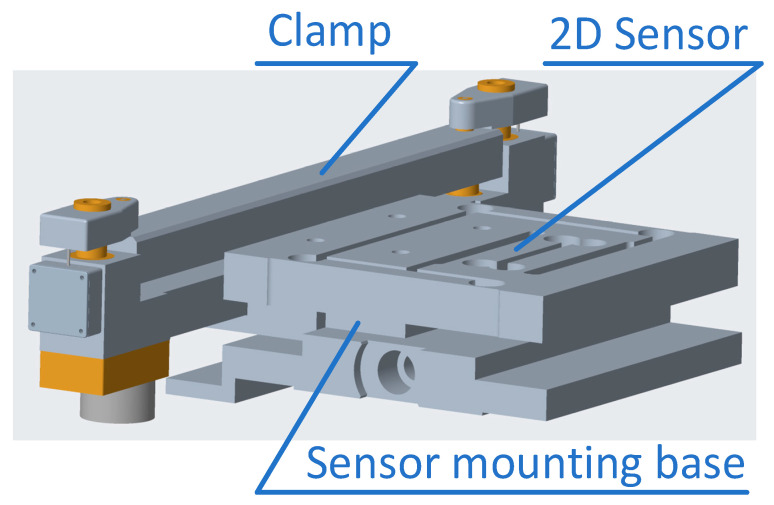
Schematic diagram of sensor installation relationship. In the figure, the sensor is affected by lateral forces due to the gravity of the clamp.

Based on the above analysis, considering the specific needs of fabric measurement, the elastic element of the fabric measurement sensor is designed as shown in Figure 9. In Figure 9, the structure of the tensile measurement sensor elastic element is designed as an S-shaped sensor, nested in the middle of the elastic element of the shear measurement sensor. When the sensor is in a zero-force state, the intersection of the jaw line of the fabric clamp and the centre line of the fabric tensile force coincides with the planar geometric centre of the sensor elastic element. The shear sensor elastic element is also optimised to an S-type equivalent sensor. The elastic element structural arrangement considers the following aspects:

- To minimise inter-dimensional coupling between tensile and shear forces during measurement, by increasing the shear sensor elastic element’s ability to withstand tensile forces and resist inter-dimensional coupling (for highly accurate fabric measurement, the range of tensile force measurements is 10 times greater than the range of shear force measurements), as shown in Figure 9.
- To extend the dynamic performance of the sensor by increasing the structural stiffness of the sensor elastic element as much as possible.
- To reduce sensor interference caused by the weight of the fabric clamp, as shown in Figure 10.

Inter-dimensional coupling is a key issue that needs to be overcome in the research of multidimensional force sensors, involving the accuracy and reliability of the sensors. The inter-dimensional coupling of multi-dimensional force sensors refers to the phenomenon of mutual influence between different dimensions during the transformation process of the sensor. When a multi-dimensional sensor measures forces in different dimensions, errors in the measurement results may occur due to factors such as the sensor’s structure, materials, circuits, etc. These errors are called inter-dimensional couplings (interferences).

For example, our sensor can measure forces in two dimensions, tensile and shear, at the same time. When measuring the shear force, the tensile force on the sensor affects the signal output of the shear force. Then, there is an inter-dimensional coupling. Solving inter-dimensional coupling interference requires reasonable and accurate sensor structure design, strict processing error control, calibration methods, circuit implementation, and machine intelligence correction.

The relationship of the sensor and the design of its structure are the result of a comprehensive balance of various influences on the measurement accuracy and reliability of the sensor. In the KES-F measurement system, tensile force measurement and shear force measurement are completed separately by two sensors. The two clamps of the instrument are mounted on the base via two (inevitably frictional) kinematic pairs. Its advantage is that the sensors do not have to bear the gravity and operating force acting on the clamps. Its disadvantage is that it introduces uncertain friction errors in the force transmission relationship and complicates the structure of the instrument, adding to higher manufacturing costs. As shown in Figure 10, our solution uses a specially designed 2D sensor. It simplifies the instrument structure and reduces manufacturing costs, and it eliminates measurement errors caused by friction. However, the sensor must withstand the weight of a clamp linked with the sensor and the operating force on the clamp. We use control technology with feedback to change the operating force on the clamp from the external force to the internal force of the clamp, so it has no effect on the sensor. However, the installation and structure of the sensor still need to consider the influence of the clamp’s gravity.

### 3.4. Elastic Element Calculations

Before proceeding with the subsequent calculations and finite element simulations of this paper, we need to further clarify that we provide two solutions based on different application scenarios of fabric-measuring instrumentation. Two different 2D integrated measurement sensors are being used to support these two solutions. Option one is called the rapid measurement solution. It uses buckling measurement instead of pure bending measurement. Its two-dimensional integrated measuring sensors are used to measure buckling, shear, and tension. Option two is called the precision measurement solution. Its two-dimensional integrated measuring sensor is used for shear and tensile measurements. The precise measuring solution additionally comes with a purely bending measuring unit. eliminating the need to use buckling measurement. The structure and installation relationship of the two two-dimensional integrated measurement sensors are the same. They also contain the same shear measurement capabilities. However, due to the need of buckling measurement, the ranges of the tensile-dimensional sensors are not the same. The measuring range of the tensile dimension of the rapid measurement solution is 10 N and the tensile dimension of the precise measurement is 100 N. Therefore, the change in measuring range makes the inter-dimensional coupling relationship of the two sensors different.

In the following calculation, since the shear dimension measurement function, structure, and range of the two sensors are the same, they share the same calculation result $h_{s}$ of the thickness of the sensitive position. However, the sensitive location thicknesses of their tensile dimensions are calculated for $h_{t10}$ and $h_{t100}$, respectively. In Section 3, the elastic elements of the two sensors are separately simulated and calculated using finite elements.

As a compromise between the sensitivity and rigidity of the sensor, the nominal sensitivity coefficient of the sensor is selected as *K* = 1.5 mV/V. Since the strain utilisation factor is less than 1, the actual sensitivity factor of the sensor can be between 1 mV/V and 1.5 mV/V. Let us consider the sensitivity coefficient of the strain gauge as k=2. According to the structural design of the sensor, the centre distance of the strain gauge of the tensile sensor is 50 mm. The strain gauge centre distance of the shear sensor is 68 mm. For high-accuracy fabric measurement, the measured upper range of the tensile force is 100 Newtons, and that of the shear force 10 Newtons. For rapid measurement, the tensile and shear forces are 10 N. The Young’s modulus of aluminum-copper alloy is E = 73.1 GPa. 

Substituting the *K* and *k* values into Equation (9), the strain value $\varepsilon_{m}$ at the sensitive position of the sensor can be obtained:

$$\varepsilon_{m}=\frac{K}{k}=\frac{1.5\;*\;10^{-3}}{2}=750\;*\;10^{-6}$$

The section modulus of the sensor sensitive location is as follows:

(11) $$\begin{array}{c} W=\frac{bh^{2}}{6} \end{array}$$

Substituting Equation (11) into Equation (7), we can obtain:

(12) $$\begin{array}{c} \varepsilon_{m}=\frac{Pa}{2EW}=\frac{3Pa}{Ebh^{2}} \end{array}$$

Therefore, the thickness h of the sensor sensitive location can be obtained:

(13) $$\begin{array}{c} h=\sqrt{\frac{3Pa}{b{E\varepsilon }_{m}}} \end{array}$$

Use Equation (13) to calculate the thickness $h_{s}$ of the shear measurement sensitive position of the two two-dimensional sensors, and the maximum value of the shear measurement force P=10 N. It can be seen from Figure 9 that half of the distance between the two sensitive holes of the shear measurement beam is a=34 [mm]. As can be seen from Figure 9, the thickness of the sensor structure is b=15 [mm]. The modulus of the sensor elastomer is E=73.1 GPa. Substituting the above values into Equation (13), we can obtain:

$$h_{s}=\sqrt{\frac{3\;*\;10\;*\;34\;*\;10^{-3}}{15\;*\;10^{-3}\;*\;73.1\;*\;10^{9}\;*\;750\;*\;10^{-6}}}=1.114\;[mm]$$

Use Equation (13) to calculate the thickness $h_{t100}$ of the tensile measurement sensitive position of the two-dimensional sensor for precision measurement solution, the maximum value of the tensile force measurement range P=100 N, and a=25 [mm]. Substituting into Equation (13), we obtain:

$$h_{t100}=\sqrt{\frac{3\;*\;100\;*\;25\;*\;10^{-3}}{15\;*\;10^{-3}\;*\;73.1\;*\;10^{9}\;*\;750\;*\;10^{-6}}}=3.02\;[mm]$$

Similarly, use Equation (13) to calculate the thickness $h_{t10}$ of the tensile measurement sensitive position of the two-dimensional sensor for rapid measurement solution, the maximum value of the tensile force measurement range P=10 N, and a=25 [mm]. Substituting into Equation (13), we obtain:

$$h_{t10}=\sqrt{\frac{3\;*\;10\;*\;25\;*\;10^{-3}}{15\;*\;10^{-3}\;*\;73.1\;*\;10^{9}\;*\;750\;*\;10^{-6}}}=0.955\;\left[mm\right]$$

**Figure 11:**
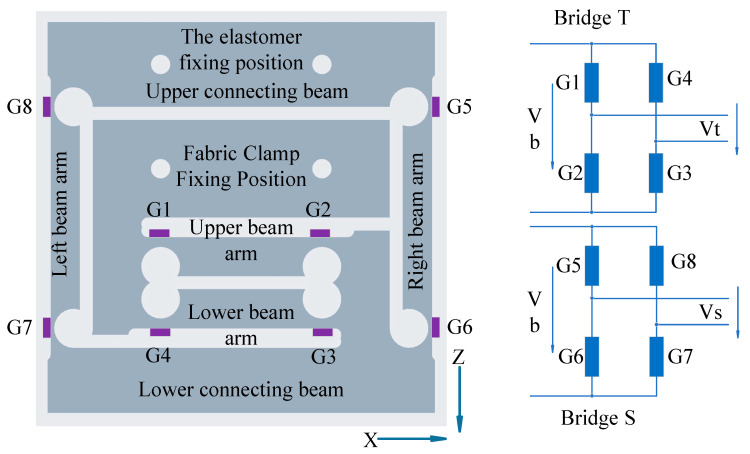
Schematic diagram of integrated multi-dimensional sensor elastic element parts nomenclature and strain gauge bridge.

**Figure 12:**
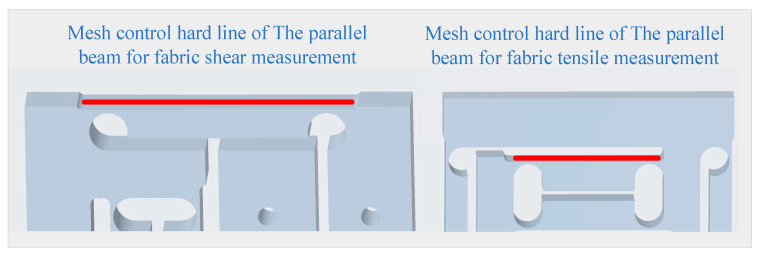
Both two-dimensional integrated sensor elastic elements mesh control hard line diagram.

**Figure 13:**
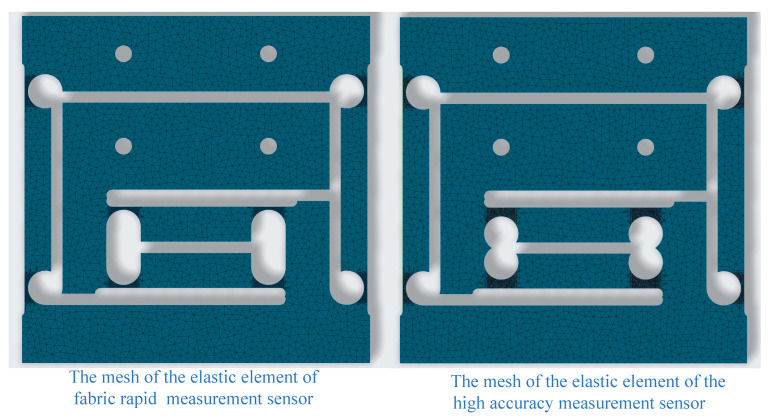
The meshing of two two-dimensional integrated sensors for the precise measurement solution and for the rapid measurement solution.

Applying the above calculation results to the geometric dimensions of the two two-dimensional sensor elastic elements and meshing them, the two elastic elements shown in Figure 11, Figure 12 and Figure 13 can be obtained. Using finite element calculations, sensor design effects can be simulated and verified.

## 4. Finite Element Simulation of Sensor Elastic Elements

### 4.1. Integrated Multi-Dimensional Sensor Elastic Elements: Parts and Strain Gauges Bridging

Both two-dimensional sensor elastic elements are integrated with two parallel beam sensors. Both parallel beam sensors are S-type sensors and are being optimised. The sensor parallel beam elastic elements for measuring fabric tensile force consists of an upper and a lower beam arm. Strain gauges G1 to G4 are attached to their outer surfaces. The parallel beam elastic element of the fabric shear force sensor consists of a left and a right beam arm. Strain gauges G5 to G8 are attached to their surfaces, respectively. Sensor elastic elements share a low link beam. The upper connecting beam is used to secure the sensor to the instrument frame. The fabric clamp is fixed to the upper part of the S-type sensor’s elastic element used to measure the tensile force of the fabric. It forms a three-dimensional force transmission chain of fabric tensile force, shear force, and the gravity of the fabric clamp between the clamp and the instrument frame.

G1 to G4 are connected to form a Wheatstone bridge for fabric tensile force measurement. G5 to G8 are connected to form a Wheatstone bridge for fabric shear force measurement, as shown in Figure 11.

Let the resistance of all strain gauges be *R* and refer to Equation (8). For bridge T, the voltage at the midpoint of the two half-bridges are:

$$V_{t1}=\frac{R+∆R_{2}}{R+∆R_{1}+R+∆R_{2}}V_{b}=\frac{1+\frac{∆R_{2}}{R}}{2+(\frac{∆R_{1}}{R}+\frac{∆R_{2}}{R})}V_{b}=\frac{1+k\varepsilon_{G2}}{2+k(\varepsilon_{G1}+\varepsilon_{G2})}V_{b}$$

and:

$$V_{t2}=\frac{1+k\varepsilon_{G3}}{2+k(\varepsilon_{G3}+\varepsilon_{G4})}V_{b}$$

The output *Vt* of bridge *T* is:

(14) $$\begin{array}{c} V_{t}=V_{t1}-V_{t2}=\left(\frac{1+k\varepsilon_{G2}}{2+k\left(\varepsilon_{G1}+\varepsilon_{G2}\right)}-\frac{1+k\varepsilon_{G3}}{2+k\left(\varepsilon_{G3}+\varepsilon_{G4}\right)}\right)*V_{b} \end{array}$$

The sensitivity $K_{t}$ of the bridge *T* is:

(15) $$\begin{array}{c} K_{t}=\frac{V_{t1}-V_{t2}}{V_{b}}=\frac{1+k\varepsilon_{G2}}{2+k\left(\varepsilon_{G1}+\varepsilon_{G2}\right)}-\frac{1+k\varepsilon_{G3}}{2+k\left(\varepsilon_{G3}+\varepsilon_{G4}\right)} \end{array}$$

The output *Vs* of bridge *S* is:

(16) $$\begin{array}{c} V_{s}=V_{s1}-V_{s2}=\left(\frac{1+k\varepsilon_{G6}}{2+k\left(\varepsilon_{G5}+\varepsilon_{G6}\right)}-\frac{1+k\varepsilon_{G7}}{2+k\left(\varepsilon_{G7}+\varepsilon_{G8}\right)}\right)*V_{b} \end{array}$$

The sensitivity $K_{s}$ of the bridge *S* is:

(17) $$\begin{array}{c} K_{s}=\frac{V_{s1}-V_{s2}}{V_{b}}=\frac{1+k\varepsilon_{G6}}{2+k\left(\varepsilon_{G5}+\varepsilon_{G6}\right)}-\frac{1+k\varepsilon_{G7}}{2+k\left(\varepsilon_{G7}+\varepsilon_{G8}\right)} \end{array}$$

In Equations (14)–(17), $\varepsilon_{Gi}\;(i=1\;to\;8)$ is the strain value of the elastic element at the position of corresponding numbered strain gauge.

### 4.2. Mesh Control and Generation

In the process of finite element analysis, mesh division plays an important role in the simulation process and the accuracy of the results obtained by the calculation. To obtain accurate calculation results and reduce the computational complexity of the simulation process as much as possible, the method of sub-regional control of the side length of the mesh is adopted. After several attempts, the maximum element size of the elastic element mesh in the main deformation region was limited to 0.4 mm. In other areas, the maximum element size of the elastic element mesh is limited to 3 mm. 

To facilitate the observation of the stress/strain state of the parallel beam surfaces, hard lines are placed on the surfaces of the left, right, upper, and lower beam arms of the elastic element to control the generation of meshes. The hard lines setup is shown in Figure 12. 

The meshing of two two-dimensional integrated sensors for the precise measurement solution and for the rapid measurement solution is shown in Figure 13. It is worth noting that the hardwired setup is the same for both two-dimensional integrated sensors.

### 4.3. Simulation Results and Analysis of Elastic Element for the Rapid Measurement Solution

#### 4.3.1. The Simulation Analysis (Tensile Simulation) of the Elastic Element for Rapid Measurement Solution

**Figure 14:**
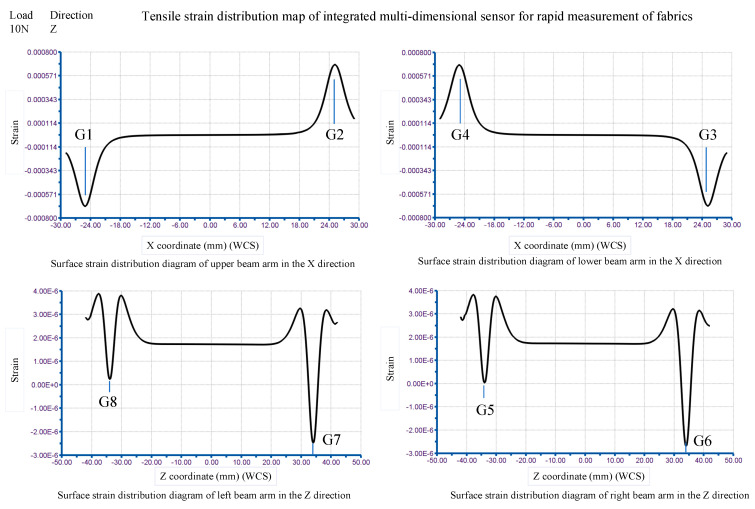
Strain distribution diagram of the elastic element of the sensor for fast measurement solutions (tensile simulation).

At the position of the jaw line of the fabric clamp, in the z-direction, a force of 10 N is applied to the fabric rapid measurement sensor, and the finite element simulation results shown in Figure 14 have been obtained. The figure shows the strain distribution on the surfaces of the four strain beam arms. They are based on the corresponding four reference hard lines.

The strain grid length for the strain gauge was chosen to be 2.5 mm. The centre distance between G1 (G4) and G2 (G3) is 50 mm. They are symmetrical about the centerline of the sensor. The centre distance between G5 (G8) and G6 (G7) is 68 mm. They are also symmetrical to the other midline of the sensor. By acquiring the data for the four curves in Figure 13, the elastic element representation strain data for the range covered by the strain grid length are extracted at the eight strain gauge locations. The obtained results are shown in Table 1 and Table 2.

Since the strain gauge sensitivity k=2 and the Wheatstone bridge excitation voltage $V_{b}=5\;V$, substituting the corresponding average strain value obtained by simulation into Equation (15), the sensitivity $K_{t10}$ of the bridge *T* can be obtained as:

$$K_{t10}=1.2606\;*\;10^{-3}$$

Bridge *T* output voltage $V_{t10}=K_{t10}\;*\;V_{b}=5\;*\;1.2606\;*\;10^{-3}=6.303\;[mV]$.

Similarly, substituting the corresponding average strain value obtained by simulations into Equation (16), the output $V_{ts10}$ of the bridge *S* is:

$$V_{ts10}=-0.054\;[mV]$$

It is worth noting that, in this simulation, no external force is applied to the sensor in the x-direction and the output of the bridge S comes from the inter-dimensional coupling of the sensor. That is, the tensile force is incorrectly coupled to the output of the shear sensor. Artificial intelligence decoupling algorithms will be used to remove the inter-dimensional coupling.

#### 4.3.2. The Simulation Analysis (Shear Simulation) of the Elastic Element for Rapid Measurement Solution

**Figure 15:**
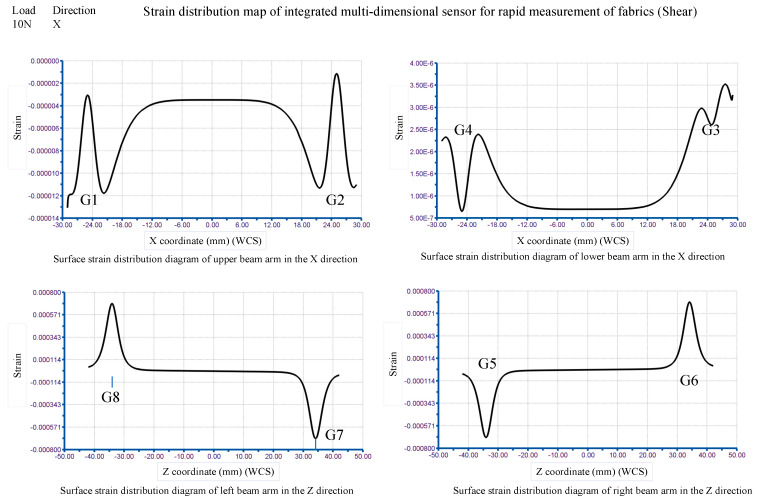
Strain distribution diagram of the elastic element of the sensor for fast measurement solutions (shear simulation).

At the position of the fabric clamp jaw line, in the x-direction, a force of 10 N is applied to the rapid measurement sensor and the results of the finite element simulation shown in Figure 15 have been obtained. The figure shows the stress distribution on the surfaces of the four strain beam arms. They are based on the corresponding four reference hard lines.

The data of the four curves are shown in Figure 15 and the strain data of the elastic element surface covering the length of the strain grid at the positions of the eight strain gauges are being obtained. The data-processing results are shown in Table 3 and Table 4. The sensitivity of the bridge S can be calculated from the data in Table 3 and Table 4.

Substituting the corresponding average strain value obtained by simulation into Equation (17), the sensitivity $K_{s10}$ of the bridge S is:

$$K_{s10}=1.2869\;*\;10^{-3}$$

The output voltage $V_{s10}$ of bridge S is:

$$V_{s10}=1.2869\;*\;5=6.4345\;[\mathrm{mV}]$$

Substituting the corresponding average strain value obtained by simulation into Equation (14), the output voltage $V_{st10}$ of bridge *T* is:

$$V_{st10}=\;-0.5098\;[\mathrm{mV}]$$

The output $V_{st10}$ is interdimensional coupling and must be operated by decoupling.

#### 4.3.3. Simulation Analysis of Elastic Elements Displacements

**Figure 16:**
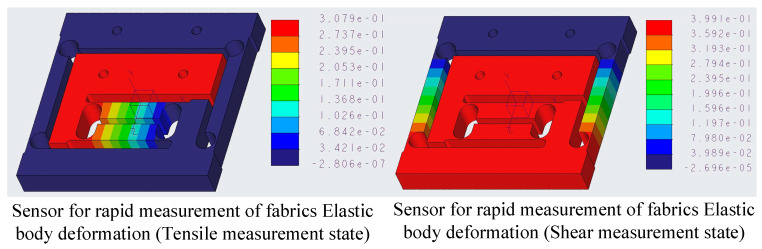
Simulation cloud of handy measuring sensor elastic elements deformation.

As can be observed in Figure 16, the sensor elastic element undergoes a displacement of 0.3079 mm in the tensile measurement direction and 0.3991 mm in the shear measurement direction, respectively, under a force of 10 N in the corresponding direction.

### 4.4. Summary of Sensor Simulation Results

With the same simulation and calculation process, the data from the accurate measuring sensor can be obtained. The results of the two sensor simulations are summarised in Table 5.

The simulation results show that the strain in positions G1 to G8 is appropriate for both sensors’ elastic element. The strain utilisation factors are within a reasonable range. The bridge sensitivity and output voltage range are also in line with design expectations.

As both are multi-dimensional sensors, there is a significant coupling between dimensions. If the sensor is used in the traditional way of measuring fabrics, i.e., in a single dimension, the inter-dimensional coupling does not affect the accuracy of measurement. However, if the sensor is used for multidimensional simultaneous measurements, the coupling needs to be decoupled with the help of virtual sensor technology and artificial intelligence techniques, such as LS-SVM.

The simulation results show that there is a significant deformation of the sensor’s elastic element during the measurement process. It inevitably leads to an additional deflection from the position of the fabric clamp. On the one hand, it is unavoidable for any sensor that uses the deformation of an elastic element as the principle of force measurement. On the other hand, it does cause errors in the measurement of the fabric. Most existing force sensors are based on the elastic deformation principle; force sensors using elastic ring deformation plus differential transformers are also included. Errors are often overlooked. There are two ways to resolve errors caused by displacement. One way is to increase the rigidity of the sensor elastic element as much as possible. But this is not desirable because it will result in a weaker output signal from the sensor, and it will not eliminate the error entirely. The other way is for the computer to identify and compensate for errors. It can fundamentally eliminate the influence of position bias on measurement accuracy. The sensor elastic element works in a good linear deformation range. Errors resulting from the movement of the position of the part of the elastic element holding the fabric clamp can be removed by calculation in the virtual sensor module. It is also necessary to compensate for the positioning of the motion of the drive motor. Compensation of the target positioning of the drive motor movement can be achieved by the real-time predictive control of the system during the measurement process.

In the next section, the paper introduces the design of the analogue front end of the sensor module, especially the design of the analogue signal conditioning circuit with low offset, low temperature drift, and low power consumption, and its suitability for the dynamic measurement of fabric tensile, shear, and buckling.

## 5. Analogue Front Ends and Sensor Signal Conditioning

The design of the analogue front-end hardware depends on the functionality it needs to provide. It is a compromise result of function and task distribution and co-operation between hardware and software implementation. Properly designed hardware resources are important for effective software processing. However, important functions should be implemented in the software, as much as possible. The reason for this is:

- Once the software is developed and debugged, no additional costs are incurred during the manufacturing process and during use.
- The software does not have problems caused by temperature drift, etc.
- There is no inconsistency in the information processing of the software.
- After the making of the instrument is complete, the software can be easily updated as needed.
- Software has no environmental influence.

In the traditional sensor-manufacturing process, the zero-point sensitivity, temperature sensitivity, coefficient adjustments, nonlinear correction, and other operations of the sensor occupy a lot of resources and time. It makes the cost of the sensor increase, and, despite all of that, it can still not work well. In the integrated sensor module, these tasks will be handed over to the firmware as much as possible. However, the hardware system, including the host microcontroller, must provide adequate support for software processing.

The analogue front end with integrated sensors is planned, together with the design of the analogue front end of the entire measurement system. The integrated sensor function block occupies two signal-processing channels in the analogue front end of the entire measurement system. For a comprehensive fabric measurement system, two additional channels are required for other measurement functions. Therefore, the analogue front end of the system needs to have four signal-processing channels.

To further improve the accuracy of the measurement system, the virtual sensor module needs to decouple the tensile and shear information that includes the interdimensional coupling. The information of these two channels must be obtained by simultaneous sampling. Moreover, to reduce the overall measurement time, some measurements need to be performed simultaneously. The analogue front-end information channel must be capable of parallel synchronous sampling and information processing.

Systems typically need to provide an effective digital resolution of 1/20000 of mechanical information. They need to occupy a 15-bit width. However, if corrections such as sensor and circuit offset zeroing, temperature drift removal, etc. are left to software to complete, the system will require a much higher bit width. Therefore, usually, a width of 24 bits is used, which is also what we have used.

The measurement of fabric mechanical properties is a dynamic process. The force on the sensor changes dynamically during the measurement process. Unlike strain-weighing measurement systems, fabric force measurement systems require dynamic measurement capabilities.

Low-cost, high-bit-width ADC chips usually use the Delta-Sigma architecture. ADCs that use oversampling techniques will simplify the design of anti-aliasing analogue low-pass filters. However, since the ADC uses a digital filter to process the output information stream, it is not conducive to quickly time-multiplexing the ADC between different measurement channels. 

Considering the above requirements, Texas Instruments’ ADS1274 24-Bit, 4-Channel Simultaneous-Sampling Delta-Sigma ADC was chosen as the main chip of the system’s analogue front end [29]. To meet the system requirements and minimise system power consumption, the chip is set to work in its low-speed mode. In this mode, the analogue front end provides a channel data throughput rate of 10 kSPS and a power consumption of 7 mW/Channel. The chip adopts a linear phase digital filter, which is conducive to the synchronous processing of multi-channel information flow.

The circuit uses a 5V power supply to power the measurement bridge. The 1/2 bridge voltage is used as the reference voltage for the ADC. This arrangement is used to eliminate the disturbance of the measurement results due to changes in the measurement bridge voltage.

**Figure 17:**
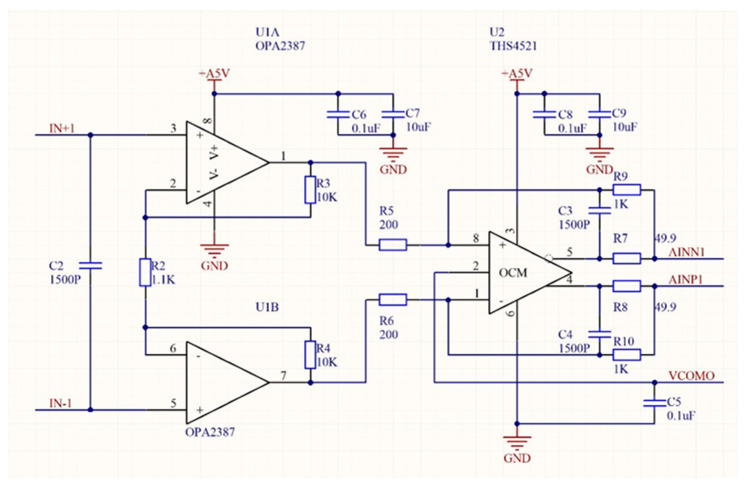
Analogue channel signal conditioning circuit diagram.

The analogue channel signal conditioning circuit is shown in Figure 17. In Figure 17, U2 is designed according to Texas Instruments’ requirements for the analogue channel driver circuit of the ADS1274 chip [29]. But, for our sensor signal, the amplification provided by U2 cannot fully utilise the dynamic input range provided by ADS1274. In this design, we added a differential amplifier composed of U1, which also greatly increases the input impedance of the entire amplifier. Hence, U1 and U2 combine to form an instrumentation amplifier. U1 is a pair of high-precision, low-offset, low-temperature-drift operational amplifiers. U1 and U2 form a fully differential amplifier. The output of the amplifier is used to drive the ADC. The circuit provides 100 times the analogue signal amplification and high common mode rejection for each measurement channel. In addition, the analogue signal conditioning circuit provides the ADC with an anti-aliasing filter function. The anti-aliasing filter is a first-order low-pass filter whose passband bandwidth is determined by R7 + R9 and C3 in Figure 17. R8 + R10 and C4 are dual elements of R7 + R9 and C3. 

The output of the analogue signal conditioning circuit is used to drive the ADC. The ADC’s control signals and output information flow are connected to the main microcontroller through the SPI interface.

At the heart of the host microcontroller system is an ARM Cortex-M33 microcontroller [30]. It works at 160 MHz main frequency, and it can provide a 240 DMIPS information processing capacity. The chip has DSP and floating-point instruction set extensions. These instruction sets are very important for sensor information processing and virtual sensor functions. The chip has an optional memory protection unit, which is especially suitable for real-time multitasking in a networking environment. The input is connected to the four uniaxial strain gauge sensors. The output is connected to the PC via USB interface. With the support of our own software developed on the .NET platform, the testing and debugging of this circuit were completed.

A software abstraction layer (virtual sensor [31]) is used to indirectly measure and compensate for the influence of the deformation of the sensor spring element on the fabric measurement accuracy. It is also used to decouple interdimensional coupling, handle nonlinear corrections, and provide temperature drift tracking.

## 6. Conclusions

A novel integrated, multi-dimensional, one-shape force sensor has been designed, capable of different measurement ranges, for obtaining accurate and rapid measurement data of fabric tensile, buckling/bending, and shear mechanics. Analysis of finite element simulation data has shown that the sensor can meet the needs of fabric tension, shear, and buckling measurements, under the ranges of 0 to 10 N, −10 N to 10 N, and 0 to 10 N, respectively. The elastic element of this sensor is based on the parallel beam elastic design due to its superior resistance to lateral force interference, made with duralumin-copper alloy. 

The results also show that the sensors have obvious inter-dimensional coupling, which requires the subsequent intelligent digital processing of inter-dimensional decoupling. The dynamic adjustment of the sensor, nonlinear, temperature, and deformation compensations also require digital processing. Hence, an analogue front-end circuit adapted to the sensor has been developed, based on a high-speed multi-channel 24-bit synchronous-sampling ADC—the TI ADS1274 24-Bit—which can meet the needs of the subsequent digital processing of the sensor’s information flow. The host microcontroller of this system is an ARM Cortex-M3, working at 160 MHz, and providing a 240 DMIPS information processing capacity, with DSP and floating-point instruction set extensions.

The proposed sensor fulfils the need for advancing the measurement of fabric mechanics under low stress by providing a stepping stone to designing and building a new rapid testing instrument adopting the advantages of current instruments, whilst eliminating their shortcomings. By doing so, it facilitates progress in CAD, home shopping, virtual reality, film, television, games, and advertising, in which fabrics and garments need to have digital twins.

## Figures and Tables

**Table 1:** Strain state of the rapid measurement sensor at strain gauge positions G1 to G4 under a load of 10 N (tensile simulation).

	G1	G2	G3	G4
Average strain value	−6.3716	6.1828	−6.3809	6.2759
Max strain value (Abs)	−6.8594	6.7779	−6.8588	6.7811
Strain utilisation factor	0.92888	0.91207	0.93032	0.92551

**Table 2:** Strain state of the rapid measurement sensor elastic element at strain gauge positions G5 to G8 under a load of 10 N (tensile simulation).

	G5	G6	G7	G8
Average strain value	5.38345 × 10^−7^	−2.00062 × 10^−6^	−1.81879 × 10^−6^	6.98684 × 10^−7^

**Table 3:** Strain state of the measurement sensor elastic element at strain gauge positions G5 to G8 under a load of 10 N (shear simulation).

	G5	G6	G7	G8
Average strain value	−6.4317 × 10^−4^	6.4484 × 10^−4^	−6.4077 × 10^−4^	6.4511 × 10^−4^
Max strain value (Abs)	−6.8919 × 10^−4^	6.9023 × 10^−4^	−6.8675 × 10^−4^	6.8841 × 10^−4^
Strain utilisation factor	0.93322	0.93424	0.93305	0.93710

**Table 4:** Strain state of the measurement elastic element at strain gauge positions G1 to G4 under a load of 10 N (shear simulation).

	G1	G2	G3	G4
Average strain value	−4.4102 × 10^−6^	−2.7738 × 10^−6^	2.7449 × 10^−6^	9.0456 × 10^−7^

**Table 5:** Summary of sensor simulation results.

	Rapid Measurement Sensor		Accurate Measurement Sensor	
	T	S	T	S
G1 strain mean	−6.3716 × 10^−4^	−4.4102 × 10^−6^	−6.7800 × 10^−4^	−2.6776 × 10^−6^
G2 strain mean	6.1828 × 10^−4^	−2.7738 × 10^−6^	6.3514 × 10^−4^	−7.1012 × 10^7^
G3 strain mean	−6.3809 × 10^−4^	2.7449 × 10^−6^	−6.7939 × 10^−4^	2.5647 × 10^−6^
G4 strain mean	6.2759 × 10^−4^	9.0456 × 10−7	6.3271 × 10^−4^	5.5698 × 10^−7^
G5 strain mean	5.3835 × 10 × 10^−7^	−64317 × 10^−4^	4.8108 × 10^−6^	−6.4972 × 10^−4^
G6 strain mean	−2.0006 × 10^−6^	6.4484 × 10^−4^	−2.0042 × 10^−5^	6.4424 × 10^−4^
G7 strain mean	−1.8188 × 10^−6^	−64077 × 10^−4^	−1.8827 × 10^−5^	−6.4492 × 10^−4^
G8 strain mean	6.9868 × 10^−7^	6.4511 × 10^−4^	7.1813 × 10^−6^	6.4039 × 10^−4^
Strain utilisation factor main	0.9242	0.9344	0.9711	0.9364
Displacement [mm]	0.3079	0.3991	0.22715	0.3986
Bridge sensitivity	1.2606 × 10^−3^	1.2869 × 10^−3^	1.3127 × 10^−3^	1.2896 × 10^−3^
Bridge output [mV]	6.303	6.4345	6.563	6.448
Interdimensional coupling [mV]	−0.054	−0.5098	−0.2136	−0.0001

## Data Availability

The data that support the findings of this study are available from the corresponding author upon reasonable request.
